# Is rectus abdominis thickness associated with survival among patients with liver cirrhosis? A prospective cohort study

**DOI:** 10.1590/1516-3180.2019.000406082019

**Published:** 2019-11-07

**Authors:** Maria Ciocîrlan, Mircea Mănuc, Mircea Diculescu, Mihai Ciocîrlan

**Affiliations:** I MD. Physician and Assistant Professor, Department of Gastroenterology and Hepatology, Fundeni Clinical Institute, Carol Davila University of Medicine and Pharmacy, Bucharest, Romania.; II MD. Physician and Associate Professor, Department of Gastroenterology and Hepatology, Fundeni Clinical Institute, Carol Davila University of Medicine and Pharmacy, Bucharest, Romania.; III MD. Physician and Professor, Department of Gastroenterology and Hepatology, Fundeni Clinical Institute, Carol Davila University of Medicine and Pharmacy, Bucharest, Romania.; IV MD. Physician and Senior Lecturer, Department of Gastroenterology and Hepatology, University of Medicine and Pharmacy, Agrippa Ionescu Clinical Emergency Hospital, Bucharest, Romania.

**Keywords:** Liver cirrhosis, Sarcopenia, Ultrasonography, Rectus abdominis

## Abstract

**BACKGROUND:**

Sarcopenia may affect patients with liver cirrhosis and worsen disease outcomes.

**OBJECTIVES:**

To evaluate ultrasound-measured psoas major (PM) and rectus abdominis (RA) thickness for predicting survival among patients with liver cirrhosis.

**DESIGN AND SETTING:**

Prospective cohort study in a tertiary-level hospital.

**METHODS:**

61 patients with liver cirrhosis were prospectively included during a 15-month period and followed up for at least six months. Cirrhosis was classified using the Child-Pugh score. Sarcopenia was assessed using surrogate parameters: handgrip strength (HGS), mid-arm muscle circumference (MAMC) and SGA (subjective global assessment). We used ultrasound to measure RA and PM thickness at admission.

**RESULTS:**

There were 41 men. The patients’ mean age was 58.03 ± 10.8 years. 26.22% of them were Child-Pugh A, 45.9% B and 27.86% C. The patients were followed up for 11.9 ± 5.63 months. RA thickness correlated moderately with MAMC (r = 0. 596; P < 0.0001) and HGS (r = 0.515; P < 0.0001) and decreased with increasing SGA class (A, 10.6 ± 2.8 mm; B, 8.3 ± 1.9 mm; C, 6.5 ± 1.9 mm; P < 0.0001). Survival at six months was independently predicted by using the model for end-stage liver disease-serum sodium score (odds ratio, OR 1.305; 95% OR confidence interval 1.083-1.572; P = 0.005). Survival during follow-up was independently predicted by RA thickness (hazard ratio, HR 0.701; 95% HR confidence interval 0.533-0.922; P = 0.011) and ascites (HR 1.876; 95% HR confidence interval 1.078-3.267; P = 0.026). PM thickness did not have any predictive value.

**CONCLUSIONS:**

As a surrogate marker of sarcopenia, RA thickness may predict survival among patients with liver cirrhosis.

## INTRODUCTION

Nutritional status is often impaired among patients with liver cirrhosis, and this results in malnutrition in more than 50% of the cases.^1^ The main feature of malnutrition comprises loss of muscle mass and altered functionality, i.e. sarcopenia.^2^ It has been reported that about 40% of patients with liver cirrhosis are sarcopenic and the percentage of such patients increases along with the severity of the disease.^3^ Studies have shown that sarcopenic cirrhotic patients are at higher risk of developing complications (refractory ascites, hepatorenal syndrome, spontaneous bacterial peritonitis or hepatic encephalopathy) and death.^3^^,^^4^^,^^5^^,^^6^

The current “gold standard” in evaluating sarcopenia is skeletal muscle mass estimation by means of computed tomography (CT) scans or magnetic resonance imaging (MRI). The “skeletal muscle index”, which takes into account all skeletal muscle groups at the level of the L3-L4 vertebrae,^3^^,^^6^^,^^7^ the “psoas muscle thickness by height”^5^^,^^7^ or the “psoas muscle index” can be measured.^8^^,^^9^^,^^10^ However, CT scans and MRI are of limited use as screening tools, as they may be expensive, time consuming (MRI) and prone to artifacts in patients with ascites (MRI)^11^ or irradiating (CT scans).^12^

Surrogate markers of sarcopenia include the Subjective Global Assessment (SGA) score and anthropometric measurements such as mid-arm muscle circumference (MAMC) and hand grip strength (HGS). SGA, MAMC and HGS show correlations with the skeletal muscle index in cirrhotic patients and in other populations.^13^^,^^14^

The prognosis for patients with liver cirrhosis is determined using several scores. The Child-Pugh score was the first one to be proposed.^15^ The model for end-stage liver disease (MELD) was implemented as a prognostic score for cirrhotic patients undergoing trans-jugular intrahepatic portal-systemic shunts, but it is currently used for prioritization of liver transplantation patients.^16^ MELD-serum sodium (MELD-Na) was subsequently developed because hyponatremia proved to be an independent factor for liver disease that correlated with complications and death.^17^

Newer prognostic models encompass sarcopenia as an independent risk factor. These models include the “MELD-sarcopenia”^3^^,^^18^ and “MELD-psoas”^7^ scores.

## OBJECTIVE

The aim of our study was to estimate the presence of sarcopenia by means of abdominal muscle ultrasound and use this measurement to predict survival among patients with liver cirrhosis.

## METHODS

### Study design, setting and ethics

This was a prospective cohort study in a tertiary-level hospital in Bucharest, Romania.

The study was conducted in accordance with the ethical standards laid down in the 1964 Declaration of Helsinki. The study protocol was approved by the Ethics Committee of the Fundeni Clinical Institute (no. 5906/3.03.2015; date of approval: March 5, 2015). Informed consent was obtained from all participants.

### Participants and setting

We evaluated all patients with liver cirrhosis who had been consecutively admitted to the 2^nd^ Department of Gastroenterology and Hepatology, Fundeni Clinical Institute, over a one-year period between March 2015 and May 2016. These patients were seen either as outpatients during scheduled control visits or as inpatients who had been admitted due to complications of liver disease (ascites, hepatorenal syndrome, spontaneous bacterial peritonitis, encephalopathy or gastrointestinal bleeding).

The exclusion criteria were the following: age < 18 years; presence of acute alcoholic hepatitis, decompensated cardiac or pulmonary disease, chronic renal failure, hepatocellular carcinoma or other malignancies, neuromuscular disorders or musculoskeletal disorders; or refusal to participate in the study.

### Variables and data collection

Demographic data were collected (age and gender), along with information on the etiology of cirrhosis and its complications. We calculated the following prognostic scores for all patients upon admission: Child-Pugh, MELD and MELD-Na.

We performed nutritional assessments on all the patients, using SGA^19^ and anthropometric measurements: triceps skinfold (TS) and mid-arm circumference (MAC). We calculated the MAMC based on TS and MAC using the formula MAMC = MAC - 3.14*(TS).^8^ We determined the HGS of the dominant hand in all patients, using a Jamar dynamometer.

We measured the body mass index (BMI), but we did not report it because it may be overestimated in patients with ascites and edema.^1^ We did not use the CT scan or MRI examinations to estimate sarcopenia. Instead, for the skeletal muscle mass evaluation, we performed ultrasound (SonoAce X8, Samsung Medison, Seoul, Korea) to measure the thickness of two skeletal muscles: the rectus abdominis (RA), with a linear 7.5 MHz probe; and the psoas major (PM), with a 3.5 MHz convex probe.

For the RA measurement, the patients were asked to lie down in a supine position, with their legs straight, in a relaxed manner. A pillow was placed under the patient’s head. We identified the *linea alba* approximately 2 cm above the level of the umbilicus; from there, by moving the probe paramedially, we were able to view the right and left RA.^20^^,^^21^ We obtained transverse sections of the RA. We measured the thickness of each RA at about 3 cm laterally from the umbilicus, between the anterior and posterior fascial borders. Minimal pressure was applied when performing the examination and making the measurements, so that we would not compress the muscle. Measurements were made at the end of a normal expiration. We performed three consecutive measurements and recorded the highest value. Both right and left-side measurements were obtained and we used their mean value for comparison purposes (mRA).

For the PM measurement, the patients were asked to lie on each side, in an oblique position. We scanned each psoas muscle (in coronal sections) from the origin, going to the lower pole of the kidney and towards the iliac crest.^22^ The iliac crest corresponds to the L4-L5 vertebrae and is the level at which the cross-sectional area of the psoas muscle is largest.^23^ We made three measurements on each side and recorded the maximum distance that we obtained between the anterior and the posterior borders of the muscle, perpendicular to the longitudinal fibers, at a level slightly above the iliac crest. We defined this as the PM thickness. Both right-side and left-side measurements were obtained and we used their mean value for comparison purposes (mPM).

All nutritional measurements and sonographic examinations were performed by the same investigator (Maria Ciocîrlan), within 24 hours of admission.

Patients were followed up until liver transplantation, until death or for at least six months after their initial assessment. Patients who did not have subsequent admissions or check-ups at the hospital were contacted by telephone, in order to gather data on complications, survival or cause of death.

### Statistical analysis

Categorical variables were presented as absolute numbers and, in some cases, as percentages. Differences among categorical variables were tested using Fisher’s exact test for two groups.

Continuous variables were presented as means and standard deviations (SD) and, in some cases, as ranges. Differences in the means of continuous variables were tested using the Mann-Whitney U test for two groups or the Kruskal-Wallis test for more than two groups. A stem-and-leaf chart was used to comparatively present the means and their 95% confidence intervals. Correlations between two continuous variables were explored using the Pearson r coefficient.

Logistic regression was used to identify independent odds ratios (OR) and their 95% confidence intervals for the likelihood of death at six months.

A multivariate Cox’s proportional hazards regression model was used to predict survival. Hazard ratios (HR) were presented as absolute numbers and 95% confidence intervals.

P-values less than 0.05 were considered statistically significant.

The IBM SPSS Statistics 25 software was used for statistical analysis.

## RESULTS

The data on demographics, disease severity, complications, nutritional status and follow-up are presented in Table 1.

**Table 1:** Demographic, severity, nutritional status and survival data of the patients included (n = 61)

	Number of patients
Sex ratio	
males/females	41/20
Age, mean ± SD, years	58.03 ± 10.8
Cirrhosis etiology	
alcoholic viral mixed other*	28 24 6 3
Ascites	
absent mild moderate severe^†^	11 14 20 16
Spontaneous bacterial peritonitis	5
Hepatorenal syndrome	2
Variceal hemorrhage	7
Encephalopathy grade	
0 1 2 3 4	36 17 8 0 0
Child-Pugh score	
A B C	16 28 17
SGA class	
A B C	20 32 9
	mean ± SD
Follow-up (months)	11.9 ± 5.63 (range: 2-28)
MELD score	12.23 ± 3.53
MELD-Na score	13.72 ± 4.54

SD = standard deviation; MELD = Model For End-Stage Liver Disease; SGA = subjective global assessment.

^*^one patient with autoimmune disease, one patient with hemochromatosis and one patient with Budd-Chiari syndrome. ^†^ascites as in SGA classification (0 - absent, 1 - mild, 2 - moderate, 3 - severe).

We did not find any significant differences in mean thickness between the right and left PM (P = 0.70), or between the right and left RA (P = 0.93). There was a moderate correlation between mRA and mPM (r = 0.46, P = 0.001).

The mRA and mPM thicknesses were significantly greater in men than in women (mRA men 9.1 ± 2.5 mm versus mRA women 7.8 ± 2 mm, P = 0.021; and mPM men 27.7 ± 5.7 mm versus mPM women 24.8 ± 3.5 mm, P = 0.049). There were no sex-related differences in disease severity distribution (mean MELD score, mean MELD-Na score and Child-Pugh class) or mortality during the follow-up.

**Figure 1.**
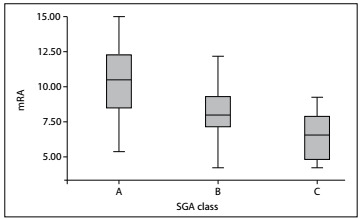
Mean rectus abdominis (mRA) measurements and their 95% confidence intervals for each subjective global assessment (SGA) class (P = 0.001).

Mean muscle thickness correlated moderately with MAMC and HGS values (for mRA and MAMC, r = 0.596, P < 0.0001; for mRA and HGS, r = 0.515, P < 0.0001; for mPM and MAMC, r = 0.323, P = 0.013; and for mPM and HGS, r = 0.496, P < 0.0001). mRA decreased significantly with progression of malnutrition, as estimated using SGA class (P = 0.001) (Figure 1). There were no significant differences in mPM among SGA classes (P = 0.071).

Out of the 61 patients, 18 died during follow-up, after a mean period of 7.2 months (range: 2-13 months). Death during follow-up occurred solely as a consequence of complications from liver cirrhosis.

Death at six months was predicted in the univariate analysis by presence of ascites (P = 0.045), mRA (P = 0.028), MELD score (P = 0.008) and MELD-Na score (P = 0.006). In the multivariate analysis, only the MELD-Na score predicted death at six months (OR 1.305; 95% OR confidence interval 1.083-1.572; P = 0.005).

Occurrence of death during the follow-up was similarly predicted in the univariate analysis by presence of ascites (P = 0.002), mRA (P = 0.001), MELD score (P = 0.015) and MELD-Na score (P = 0.013). In the multivariate analysis, only mRA (HR 0.701; 95% HR confidence interval 0.533 - 0.922; P = 0.011) and ascites (HR 1.876; 95% HR confidence interval 1.078-3.267; P = 0.026) predicted occurrence of death during the follow-up.

## DISCUSSION

Our aim was to find a simple, reproducible, noninvasive method for evaluating sarcopenia in patients with liver cirrhosis. Thus, we proposed measurement of muscle mass through ultrasound examination. Ultrasound is easy to perform, even among patients presenting difficulty in mobilization, and it is reproducible and non-irradiating. For critically ill patients, medical teams have implemented bedside ultrasound to evaluate skeletal muscle mass, with measurement of the thickness of the diaphragm or the quadriceps.^24^^,^^25^

There are no current nomograms for RA thickness in relation to age, gender, BMI and body composition. Previous studies have estimated the mean RA thickness in healthy populations, using ultrasound.^20^^,^^21^ Rankin et al. examined 123 healthy subjects (55 males) with a mean age of 40.6 ± 14.1 years. The mean values for RA thickness were significantly higher in men, with 12.5 mm ± 2.2 mm (right RA) and 12.4 ± 2.4 mm (left RA), than in women, with 10.2 ± 1.6 mm (right RA) and 10.2 ± 1.5 mm (left RA); P < 0.001.^20^ Tahan et al. conducted a study on 156 healthy subjects (75 males), with a mean age of 24.3 ± 7.2 years. They measured the thickness of RA muscles at a level similar to the level that we did. There was no significant difference between the left and right RA (P = 0.16). The mean values for RA thickness in men were 10.3 ± 1.8 mm (right RA) and 10.4 ± 1.9 mm (left RA), which were significantly greater than those in women: 8.7 ± 1.2 mm (right RA) and 8.3 ±1.3 mm (left RA); P < 0.001.^21^ We obtained similar results, but at different muscle thickness values, given that our patients had advanced liver disease and 67.2% of them presented malnutrition graded as SGA B or C.

Similarly, there are no current recommendations regarding the use of ultrasound in evaluating muscle mass and sarcopenia in patients with liver cirrhosis. Tandon et al. evaluated 159 patients with liver cirrhosis (56% male; mean age 58 ± 10 years; mean MELD 10 ± 3; 60% Child-Pugh class A) and developed a model to identify sarcopenia, using a combination of BMI and the thigh muscle thickness (AUROC 0.78 for men and 0.89 for women).^13^

Sarcopenia has already been described as an independent predictor of survival in cases of liver cirrhosis.^3^^,^^7^^,^^18^^,^^26^ Montano-Loza et al. proved that sarcopenia is an independent predictor of mortality in situations of cirrhosis and that it does not correlate with the severity of the disease as evaluated by prognostic scores (Child-Pugh and MELD).^3^ Another study conducted on a larger population (669 cirrhotic patients),^18^ proposed adding sarcopenia to MELD score (MELD-sarcopenia) for better prediction of mortality, especially among patients with MELD scores lower than 15. Sarcopenia alone would be equivalent to adding 10 points to the calculated MELD score. Similarly, Durand et al. developed a “MELD-psoas” score that could better predict mortality among patients with MELD score lower than 25 or with refractory ascites.^27^

Among our patients, although sarcopenia estimated from the RA thickness significantly predicted death at six months in univariate analysis (P = 0.028), only MELD-Na score remained an independent risk factor in multivariate analysis. However, death during follow-up (at a mean time of 11.9 months) was independently predicted from the RA thickness (HR 0.701; 95% HR confidence interval 0.533 - 0.922; P = 0.011).

PM thickness did not have any predictive value. We felt that it was more difficult to identify and accurately scan the PM muscle than the RA, using ultrasound, due to interposition of bowel gas and ascites and presence of truncal edema.

This study has several limitations. Firstly, we did not perform CT or MRI to evaluate sarcopenia. Nevertheless, very high correlations between ultrasound and MRI-measured thicknesses of RA have been reported: r = 0.932 to 0.963 in non- athletes (P < 0.001);^28^ and r = 0.847 to 0.926 in athletes (P < 0.001).^29^ Moreover, in several reports on liver cirrhosis, sarcopenia evaluated using CT or MRI has been fairly well predicted by the same surrogate markers that we used: MAMC (AUROC 0.84, P = 0.001;^13^ and r = 0.385, P < 0.001^8^), HGS (r = 0.382, P < 0.001^8^) and SGA class (in men only marginally P = 0.051;^8^ or a modified score in all patients, P < 0.001^13^).

Secondly, we did not perform multiple operator measurements or multiple measurements at different time points. Studies published previously by Tahan et al.^21^ and Wachi et al.^28^^,^^29^ also used a single operator. However, since our method for RA measurement is simple and straightforward, the expected intra-operator measurement variability would be low.^30^ In healthy adults, the standard error of measurement for RA thickness is 0.2 mm, which represents 2% of our corresponding mRA values.^31^^,^^32^

Thirdly, during follow-up, in our study cohort, only four patients were evaluated to be listed for liver transplantation. One of these was subsequently listed and successfully underwent liver transplantation. Although there are almost 6000 liver-related deaths annually in Romania and 500 to 600 patients with liver cirrhosis are listed per year, only 852 liver transplantations were performed over the last 17 years, after a mean of 20 months of waiting time on the list.^33^^,^^34^

## CONCLUSION

To the best of our knowledge, this was the first study that aimed to evaluate sarcopenia in patients with liver cirrhosis, using sonographic measurements of the RA muscles. Simple RA measurement might prove to be a useful marker for sarcopenia and prediction of survival among liver cirrhosis patients, after validation of the present results through a comparative study making correlations with the gold standard (CT or MRI), and after further refinement.
